# Reference Electrode Standardization Interpolation Technique (RESIT): A Novel Interpolation Method for Scalp EEG

**DOI:** 10.1007/s10548-021-00844-2

**Published:** 2021-05-05

**Authors:** Li Dong, Lingling Zhao, Yufan Zhang, Xue Yu, Fali Li, Jianfu Li, Yongxiu Lai, Tiejun Liu, Dezhong Yao

**Affiliations:** 1 The Clinical Hospital of Chengdu Brain Science Institute, MOE Key Lab for Neuroinformation, School of Life Science and Technology, University of Electronic Science and Technology of China, Chengdu, China; 2 Research Unit of NeuroInformation, Chinese Academy of Medical Sciences, Chengdu, 2019RU035 China; 3 School of Electrical Engineering, Zhengzhou University, Zhengzhou, 450001 China; 4 Sichuan Institute for Brain Science and Brain-Inspired Intelligence, Chengdu, 611731 China

**Keywords:** EEG, EEG preprocessing, Bad channels, Interpolation, REST reference

## Abstract

“Bad channels” are common phenomena during scalp electroencephalography (EEG) recording that arise due to various technique-related reasons, and reconstructing signals from bad channels is an inevitable choice in EEG processing. However, current interpolation methods are all based on purely mathematical interpolation theory, ignoring the neurophysiological basis of the EEG signals, and their performance needs to be further improved, especially when there are many scattered or adjacent bad channels. Therefore, a new interpolation method, named the reference electrode standardization interpolation technique (RESIT), was developed for interpolating scalp EEG channels. Resting-state and event-related EEG datasets were used to investigate the performance of the RESIT. The main results showed that (1) assuming 10% bad channels, RESIT can reconstruct the bad channels well; (2) as the percentage of bad channels increased (from 2% to 85%), the absolute and relative errors between the true and RESIT-reconstructed signals generally increased, and the correlations between the true and RESIT signals decreased; (3) for a range of bad channel percentages (2% ~ 85%), the RESIT had lower absolute error (approximately 2.39% ~ 33.5% reduction), lower relative errors (approximately 1.3% ~ 35.7% reduction) and higher correlations (approximately 2% ~ 690% increase) than traditional interpolation methods, including neighbor interpolation (NI) and spherical spline interpolation (SSI). In addition, the RESIT was integrated into the EEG preprocessing pipeline on the WeBrain cloud platform (https://webrain.uestc.edu.cn/). These results suggest that the RESIT is a promising interpolation method for both separate and simultaneous EEG preprocessing that benefits further EEG analysis, including event-related potential (ERP) analysis, EEG network analysis, and strict group-level statistics.

**Supplementary Information:**

The online version contains supplementary material available at 10.1007/s10548-021-00844-2.

## Introduction

Scalp electroencephalography (EEG) is a commonly used and excellent technique that directly quantifies the electric fields of brain activity with millisecond temporal resolution through a variable number of electrodes placed on the scalp (Cohen 2017). Since it was first reported in 1929 (Berger 1929), EEG has become one of most important cost-effective and noninvasive techniques in a wide range of fields in cognitive neuroscience (Enriquez-Geppert et al. 2017; Li et al. 2019; Tian et al. 2018), in brain-computer interfaces (He et al. 2013; Zhang et al. 2019; Zhao et al. 2020) and in the clinic (Yamada and Meng 2012). Furthermore, considering its high temporal resolution, scalp EEG provides more valuable information when fused with other popular imaging modalities, such as structure magnetic resonance imaging (sMRI) and functional MRI (Dong et al. 2014, 2015; Friston et al. 2017; Laufs 2012). In EEG practice, a common phenomenon during recordings involves some of the EEG channels being unable to accurately or correctly record the electrophysiological signals of brain activity for various technique-related reasons, including power line interference (50/60 Hz), abnormal impedance (too high or too low), broken wire contacts or other malfunctions, electrodes that are improperly placed or poorly contacted with the scalp, too much electrode jelly/paste, bridged electrodes, etc. (Hu and Zhang 2019). In these situations, these channels are usually called “bad channels”, and a simple and rough strategy is to flag these “bad channels” by hand and then remove them from further analysis. However, there are many limitations to removing “bad channels” directly. First, simply removing bad channels may be problematic. Because removing bad channels will result in loss of information, the signals from the remaining good channels may be insufficient for performing further analysis (e.g., unable to construct the full EEG network or insufficient for source imaging). Second, if the bad channels are directly removed from different subjects, the dimensions of the EEG data (channels × time points) or the good channels would change; that is, the valid channels across subjects could be different (e.g., one subject has 32 channels, while another subject has 31 channels; or subjects could have the same number of valid channels but different channel labels). Thus, this bad channel removal leads to unreasonable or non-strict group-level statistics (varied samples and degrees for each EEG channel). Third, the removal of bad channels may increase the potential risk of errors for large-scale EEG batch processing while using EEG tools (e.g., EEGLAB (Delorme and Makeig 2004) and FieldTrip (Oostenveld et al. 2011)) or cloud platforms (e.g., the WeBrain: https://webrain.uestc.edu.cn/). For these reasons, reconstructing the EEG signals of these bad channels is an alternative and inevitable approach to directly removing them.

Currently, there are several interpolation methods used to reconstruct the signals from bad channels. Because of spatial volume conduction (Yao 2000, 2017; Yao et al. 2019), the EEG signals of adjacent channels are similar. One of the first approaches developed was neighbor interpolation (NI), which can quickly reconstruct bad channels by averaging adjacent good channels (usually approximately 4 ~ 6 channels) on the scalp. Considering there are *k* (*k* < N) bad channels in N channels, the NI method can be simply formulated as

1 $$V_{NI}^{{k_{1} }} = \frac{1}{m}\left[ {\begin{array}{*{20}c} 0 & \cdots & {1_{1} } & \cdots & 0 & \cdots & {1_{m} } & \cdots & {0_{N} } \\ \end{array} } \right]V_{good}$$

where $V_{NI}^{{k_{1} }}$ represents the potentials (a vector with 1 × *T* time points) of the *k*_*1*_th interpolated channels using the NI method; *m* is the number of adjacent good channels to be averaged; and *V*_*good*_ represents the potentials of the good channels (a matrix with dimension (*N-k*) channels × *T* time points). However, the NI method is not very accurate, especially when there are too many scattered or adjacent bad channels. The most common interpolation method used in the EEG field is spherical spline interpolation (SSI) (Freeden 1984; Perrin et al. 1989). SSI mainly consists of the following steps: (1) projecting all channels onto a unit sphere; (2) calculating matrices to map the good channels to the bad channels; and (3) using the mapping matrices to estimate the interpolated EEG data of the bad channels. The SSI method can be formulated as:

2 $$\left\{ {\begin{array}{*{20}c} {G_{good} C + C_{0} = V_{good} } \\ {\sum\limits_{i = 1}^{N - k} {C_{i} } = 0} \\ {V_{SSI}^{{k_{1} }} = \sum\limits_{i = 1}^{N - k} {(C \cdot G_{sph}^{{k_{1} }} )} + c_{0} } \\ \end{array} } \right.$$

where *V*_*good*_ represents the potentials of the good channels (matrix with dimension (*N-k*) channels × *T* time points); *G*_*good*_ (a matrix with dimension (*N*-*k*) × (*N*-*k*)) represents the Legendre polynomials of the cosine of the angle between the projected good channels; *C* (a matrix with dimension (*N*-*k*) × *T*) is an unknown term that needs to be solved; *C*_*0*_ (a matrix with dimension (*N*-*k*) × *T*) is the constant of *V*_*good*_ ($$C_{0} = \left[ {\begin{array}{*{20}c} 1 \\ \vdots \\ 1 \\ \end{array} } \right]c_{0}$$, *c*_*0*_ is a vector with dimension 1 × *T*, i.e. mean of *V*_*good*_ across channels); $V_{SSI}^{{k_{1} }}$ (a vector with 1 × *T* time points) represents the potentials of the *k*_*1*_th interpolated channels using the SSI method; $G_{sph}^{{k_{1} }}$ (a matrix with dimension (*N*-*k*) channels × *T* time points) represents the Legendre polynomials of the cosine of the angle between the projected *k*_*1*_th bad channel and good channels for each time point; and “·” is the operation of multiplying corresponding elements. Due to its stability, superfast interpolation and good performance, SSI has been integrated into popular EEG tools such as EEGLAB (Delorme and Makeig 2004) and FieldTrip (Oostenveld et al. 2011) and is mainly used in preprocessing EEG signals in practice. In addition, there are some sporadic and unpopular methods, including directly inserting NaN values into bad channels (rendering them meaningless) and triangulation-based nearest neighbor interpolation both in time and space (however, the time cost of 3-D interpolation is extremely high and cannot be interrupted), which are rarely used in practical EEG preprocessing. Nevertheless, the abovementioned interpolation methods are all based on pure mathematics interpolation theory, ignoring the neurophysiological basis of the generation of EEG signals. Such interpolation methods may lead to increased errors when there are many scattered or adjacent bad channels. Furthermore, because they ignore EEG reference issues (Yao 2017; Yao et al. 2019), the performance of these interpolation methods may vary with different references, that is, it may be influenced by the choice of rereferencing methods. Therefore, a new interpolation method based on the scalp neurophysiological EEG recording model with an ideal neutral reference is required.

In this work, a new interpolation method, named the reference electrode standardization interpolation technique (RESIT), is therefore developed for the interpolation of scalp EEG channels. The paper is organized as follows. The theory and implementation of RESIT are first introduced in the next section in detail. Then, two real EEG datasets (resting-state and event-related potential data) are used to evaluate the performance of RESIT, which is then compared with the two most common interpolation methods, NI and SSI. Finally, discussions are provided regarding the performance of the proposed method.

## Material and Methods

### Reference Electrode Standardization Interpolation Technique

Here, we introduce unified mathematical representations of the reference electrode standardization interpolation technique. Considering an ideal scalp EEG recording model with a reference point at infinity, the scalp potentials *V*_*Inf*_ can be recorded with *N* channels, *M* sources, and *T* time points.

3 $$V_{Inf} = \left[ {\begin{array}{*{20}c} {v_{1}^{1} } & {v_{2}^{1} } & \cdots & {v_{T}^{1} } \\ {v_{1}^{2} } & {v_{2}^{2} } & \cdots & {v_{T}^{2} } \\ \vdots & \vdots & \ddots & \vdots \\ {v_{1}^{N} } & {v_{2}^{N} } & \cdots & {v_{T}^{N} } \\ \end{array} } \right]{\kern 1pt} \; = L_{N \times M} S_{M \times T}$$

where *L* is the lead-field matrix of size *N* × *M* determined by the head model, source model and electrode distribution; *S* is the neural source potentials with size *M* × *T* in the brain; $v_{j}^{i} ,1 \le i \le N,1 \le j \le T$ is a sample at the *j*th time point and *i*th electrode; *N* is the number of electrodes/channels; *T* is the number of time points; and *M* is the number of sources. Considering a scalp recording with reference at infinity containing *k* (*k* < N) bad channels, the scalp recording after exclusion of bad channels, $\tilde{V}_{Inf}$, can be modeled as:

4 $$\tilde{V}_{Inf} = \left[ {\begin{array}{*{20}c} {v_{1}^{1} } & {v_{2}^{1} } & \cdots & {v_{T}^{1} } \\ {v_{1}^{2} } & {v_{2}^{2} } & \cdots & {v_{T}^{2} } \\ \vdots & \vdots & \ddots & \vdots \\ {v_{1}^{N - k} } & {v_{2}^{N - k} } & \cdots & {v_{T}^{N - k} } \\ \end{array} } \right]{\kern 1pt} \; = \tilde{L}_{(N - k) \times M} S_{M \times T}$$

where $\tilde{L}$ is a lead-field matrix with the reference at infinity and excluding the bad channels. For a scalp point referenced recordings ($\tilde{V}_{e}$ with dimension (*N-k*) good channels × *T* time points) or average referenced recordings ($\tilde{V}_{AVG}$ with dimension (*N-k*) good channels × *T* time points), the scalp recording models with *k* bad channels can be expressed as:

5 $$\tilde{V}_{e} = \tilde{V}_{Inf} - wv_{e} = \tilde{L}S - wl_{e} S = (\tilde{L} - wl_{e} )S = \tilde{L}_{e} S$$

6 $$\tilde{V}_{AVG} = \tilde{V}_{Inf} - wv_{AVG} = \tilde{L}S - \frac{1}{N - k}ww^{T} \tilde{L}S = (\tilde{L} - \frac{1}{N - k}ww^{T} \tilde{L})S = \tilde{L}_{AVG} S$$

where *w* is a column vector ((*N-k)* × 1) with each of its elements being unity, i.e. $$\left[ {\begin{array}{*{20}c} 1 \\ \vdots \\ 1 \\ \end{array} } \right]$$; *S* is the neural source potentials with size *M* × *T*; *v*_*e*_ is a row vector (1 × *T*) in *V*_*Inf*_ corresponding to a scalp point reference; *l*_*e*_ is the row vector (1 × *M*) in $\tilde{L}$ ((*N-k*) × *M*) corresponding to the reference electrode; $\tilde{L}_{e}$ is a lead-field matrix ((*N-k*) × *M*) with a scalp point reference excluding the bad channels; *v*_*AVG*_ is a row vector (1 × *T*) in *V*_*Inf*_ corresponding to the average reference; $\tilde{L}_{AVG}$ is a lead-field matrix ((*N-k*) × *M*) with the average reference; *N* is the number of all channels; and *k* is the number of bad channels.

Based on the equivalent source technique (Yao 2000), the neural source potentials *S* in the brain are the same, and the use of the reference does not influence the source localization (Geselowitz 1998; Pascualmarqui and Lehmann 1993). Then, *S* can be estimated by the scalp EEG potentials excluding the bad channels:

7 $$S \approx \hat{S} = \tilde{L}_{M \times (N - k)}^{ + } \tilde{V}_{Inf} = \tilde{L}_{e}^{ + } \tilde{V}_{e} = \tilde{L}_{AVG}^{ + } \tilde{V}_{AVG}$$

where $\tilde{L}^{ + }$, $\tilde{L}_{e}^{ + }$ and $\tilde{L}_{AVG}^{ + }$ (with size *M* × (*N-k*)) are the Moore–Penrose generalized inverses of matrices $\tilde{L}$, $\tilde{L}_{e}$ and $\tilde{L}_{AVG}$, respectively; and $\hat{S}$ is the estimation of the reconstructed equivalent sources using the scalp potentials excluding the bad channels. Then, combining Eqs. (3) and (7), we can forward the estimated source potentials to all scalp electrodes (along with the bad channels) and reconstruct all potentials with the infinity reference:

8 $$\begin{gathered} V_{Inf} = LS \approx \hat{V}_{Inf} = L\hat{S} = L\tilde{L}^{ + } \tilde{V}_{Inf} \hfill \\ V_{Inf} = LS \approx \hat{V}_{Inf} = L\hat{S} = L\tilde{L}^{ + }_{e} \tilde{V}_{e} \hfill \\ V_{Inf} = LS \approx \hat{V}_{Inf} = L\hat{S} = L\tilde{L}^{ + }_{AVG} \tilde{V}_{AVG} \hfill \\ \end{gathered}$$

where $\hat{V}_{Inf}$ represents the reconstructed potentials with reference at infinity; $\hat{S}$ is the estimation of reconstructed equivalent sources; $\tilde{V}_{Inf}$, $\tilde{V}_{e}$ and $\tilde{V}_{AVG}$ (of size (*N-k*) × *T*) are the scalp EEG recordings excluding the bad channels with references, respectively, at infinity, at a scalp point and at the average reference recording; $\tilde{L}^{ + }$, $\tilde{L}_{e}^{ + }$ and $\tilde{L}_{AVG}^{ + }$ (with size *M* × (*N-k*)) are the Moore–Penrose generalized inverses of matrices $\tilde{L}$, $\tilde{L}_{e}$ and $\tilde{L}_{AVG}$, respectively, excluding the bad channels; and *L* is the lead-field matrix of size *N* × *M*. Note that if *k* = 0 (i.e., there are no bad channels), the estimated potentials $\hat{V}_{Inf}$ are equivalent to the scalp EEG signals with an infinity reference realized by REST (Dong et al. 2017; Yao 2001; Yao et al. 2005).

### Algorithm and Implementation of RESIT

RESIT consists of the following steps.

1. As an example, the coordinates of all EEG electrodes and the scalp recordings (with average reference) excluding the bad channels, $\tilde{V}_{AVG}$, are given first.
2. A three-concentric sphere head model is used for the RESIT according to previous papers (Dong et al. 2017; Yao 2001; Yao et al. 2005). The radii (normalized by the radius of the head) of the three concentric spheres are 0.87 (inner radius of the skull), 0.92 (outer radius of the skull), and 1.0 (radius of the head), and the relative conductivities are 1.0 (brain and scalp) and 0.0125 (skull) (Rush and Driscoll 1969). Then, a closed surface (such that all sources are inside the brain) is formed by a spherical cap surface (normalized by the radius of the head) with radius r = 0.869 and a transverse plane at z = -0.076, and implemented in the equivalent source model. There are a total of 3000 equivalent sources (2600 radial dipoles on the spherical cap surface and 400 dipoles (along the + z axis) on the plane).
3. The electrode coordinates are normalized and distributed on the upper spherical cap of the head model. Based on the three-concentric sphere head model, normalized electrode coordinates and equivalent source model, the lead-field matrices L in Eq. (3) and $\tilde{L}_{AVG}$ in Eq. (6) are calculated by using the forward theory of the spherical harmonic spectra (Yao 2000); then, the general inverse $\tilde{L}_{AVG}^{ + }$ of matrix $\tilde{L}_{AVG}$ is calculated.
4. For the known L, $\tilde{L}_{AVG}^{ + }$ and $\tilde{V}_{AVG}$, the final reconstructed EEG recordings $\hat{V}_{Inf}$ can be calculated according to Eq. (8).

### Real Datasets

#### Participants and Experiment

A total of 41 right-handed healthy adults (mean age = 23.9 years ± 1.6 years, age range = 21 ~ 28 years, 32 males/9 females) were recruited. All participants provided written informed consent in line with the Declaration of Helsinki before the experiment. The experiment was approved by the local Ethics Committee of the University of Electronic Science and Technology of China. For dataset 1, EEG data were recorded during the resting state (eyes-closed) for 4 min. For dataset 2, event-related potential (ERP) data were collected during a classical visual oddball P300 task, which consisted of a 1-min break and a 337.5-s task ((120 standard trials + 30 target trials) × 2.25 s). During the experiment, a bold cross was first presented for 250 ms to note the participant’s fixation point on the monitor, and then a thin cross was presented for 500 ms to inform the participant to concentrate their attention on the upcoming target or standard stimulus. The stimulus lasted 500 ms and ended with a 1000 ms break. A total of 30 target trials were collected during the experiment. All participants were instructed to omit the standard stimuli (upward-oriented triangle) and to count the number of target stimuli (downward-oriented triangle). More details of the experiment can be found in related article (Li et al. 2015).

#### EEG Recording

**Fig. 1:**
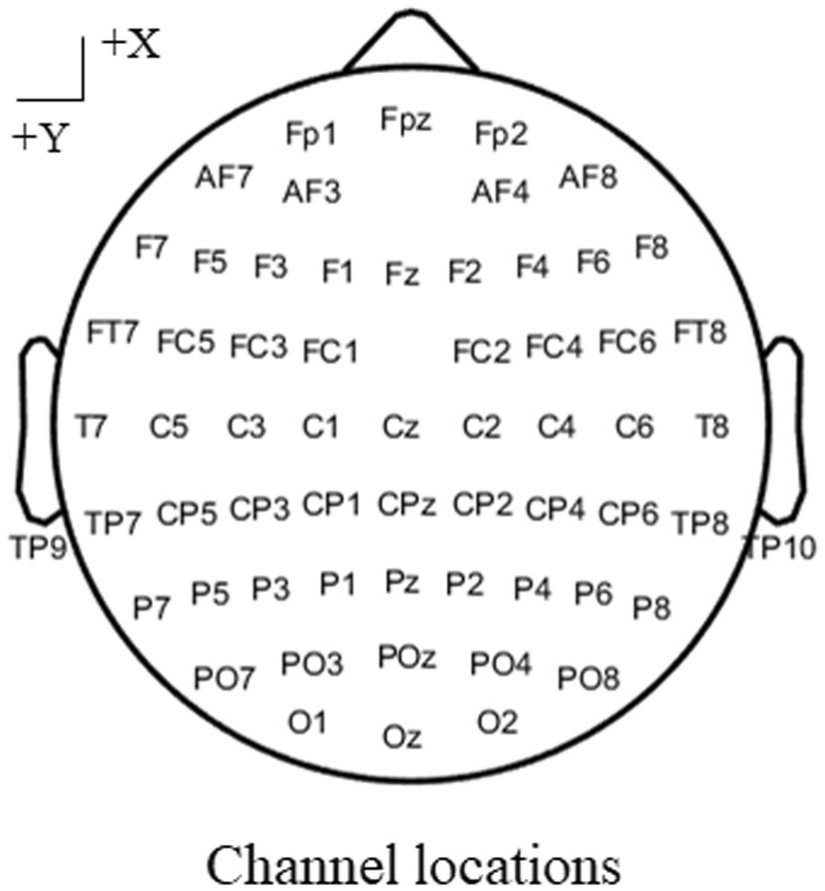
Channel locations of the EEG system. Sixty-two EEG electrodes were distributed using the international extended 10–20 cap system

The resting-state and P300-task EEG data were recorded using a 64-channel EEG system (Brain Products GmbH, Gilching, Germany). A total of sixty-two EEG electrodes were distributed on the scalp based on the international extended 10–20 cap system (Fig. 1), and 2 additional EOG channels were used to record the vertical and horizontal EOG data. The original recording reference was FCz, and the sampling rate was 500 Hz. EEG data were online bandpass filtered (0.01–100 Hz), and the impedance was maintained below 5 kΩ.

### EEG Data Preprocessing

For dataset 1, quality assessment (QA) was first conducted on the raw resting-state EEG data to detect and reject bad channels and blocks using the QA tool from the WeBrain platform (https://webrain.uestc.edu.cn/). Next, the raw data were bandpass filtered (1–30 Hz) and rereferenced to the average (AVG), and then continuous clean EEG data (>10 s) were extracted from the preprocessed data.

For dataset 2, quality assessment (QA) was also conducted on the task-related EEG data (trial level) to detect and reject bad channels. Next, the ERP analysis tool from the WeBrain platform (https://webrain.uestc.edu.cn/) was used to process the raw data, including bandpass filtering (1–30 Hz), rereferencing (AVG), data segmentation (−200 ms ~ 800 ms), baseline correction (-200 ms ~ 0 ms) and exclusion of artifact-containing trials (absolute voltage ≥ 100 μV, voltage step/sampling point ≥ 50 μV, respectively, or maximum absolute difference ≥ 150 μV). Finally, epoched and clean ERP data were obtained.

### Method Assessment

To assess the interpolation methods, two cases with bad channels were assumed. In case 1, a number of scattered bad channels (ranging from 2% to 85%) were randomly (uniform distribution) selected from clean scalp EEG data. In case 2, a set of adjacent bad channels (ranging from 2% to 85%) were randomly (uniform distribution) selected from clean scalp EEG data. Then, the performances of the interpolation methods were quantified using the mean absolute error (as well as the relative absolute error) between the interpolated EEG signals and the true signals. The mean Pearson’s correlations between the interpolated and true signals were also calculated to quantify the performances of the methods. The absolute error, relative absolute error and correlation (R) were averaged over 20 repeats of the abovementioned procedures. To assess the performances of the RESIT, the results were compared with those of SSI and NI (using the mean of 4 neighborhood channels) using one-way repeated ANOVA (*p* < 0.01) and post hoc paired t-test (*p* < 0.005). Note that the results of the RESIT were re-referenced to AVG to compare them with the results of NI and SSI, and for dataset 2 (P300 data), the indices of the errors and correlations were calculated on the averaged ERPs for each subject.

## Results

### Resting-State Data

**Fig. 2:**
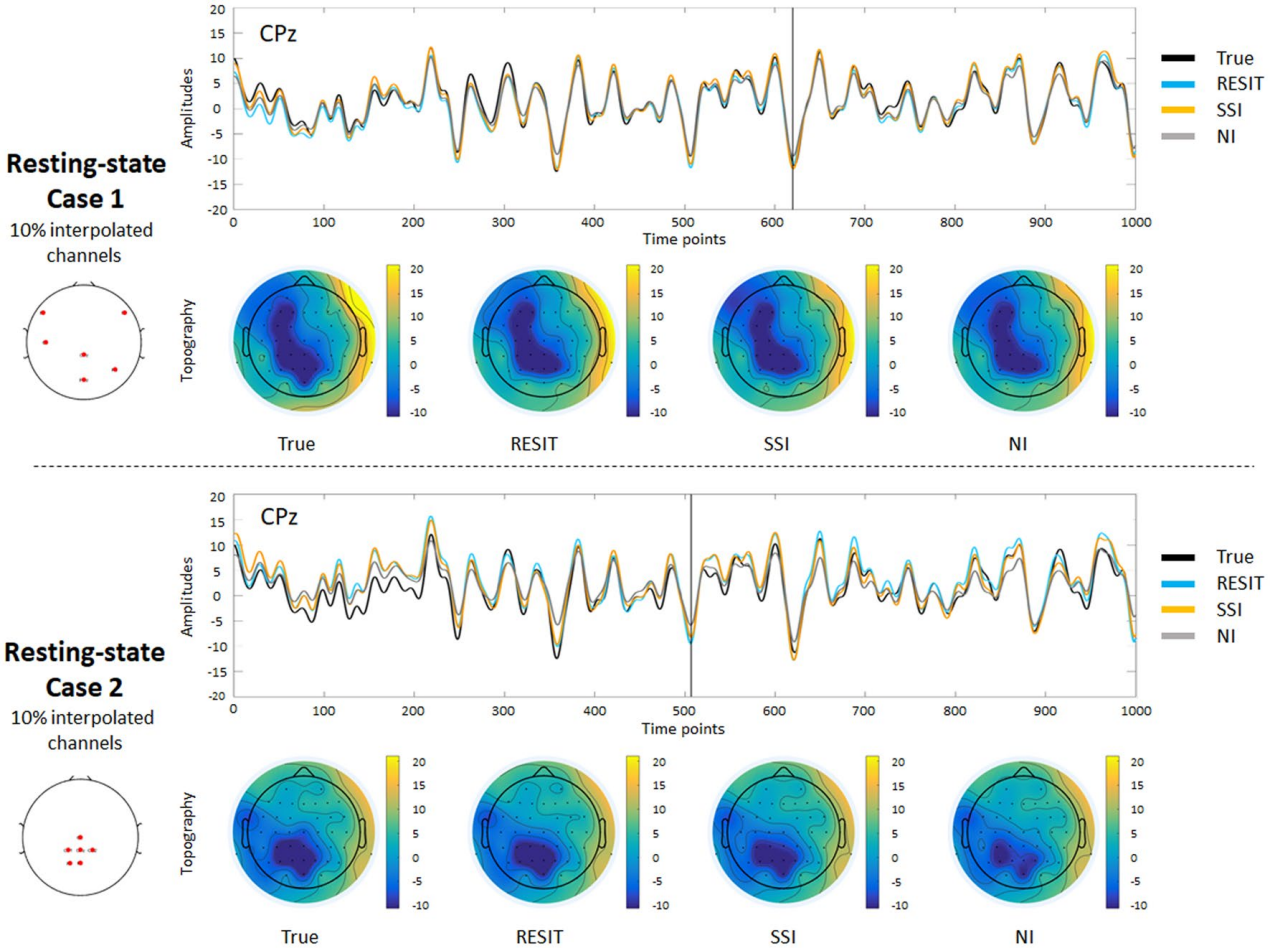
Results of different interpolation methods for resting-state EEG data from one example subject. In case 1, a number of scattered bad channels (10%) were randomly established, and in case 2, a set of adjacent bad channels (10%) was randomly established

**Fig. 3:**
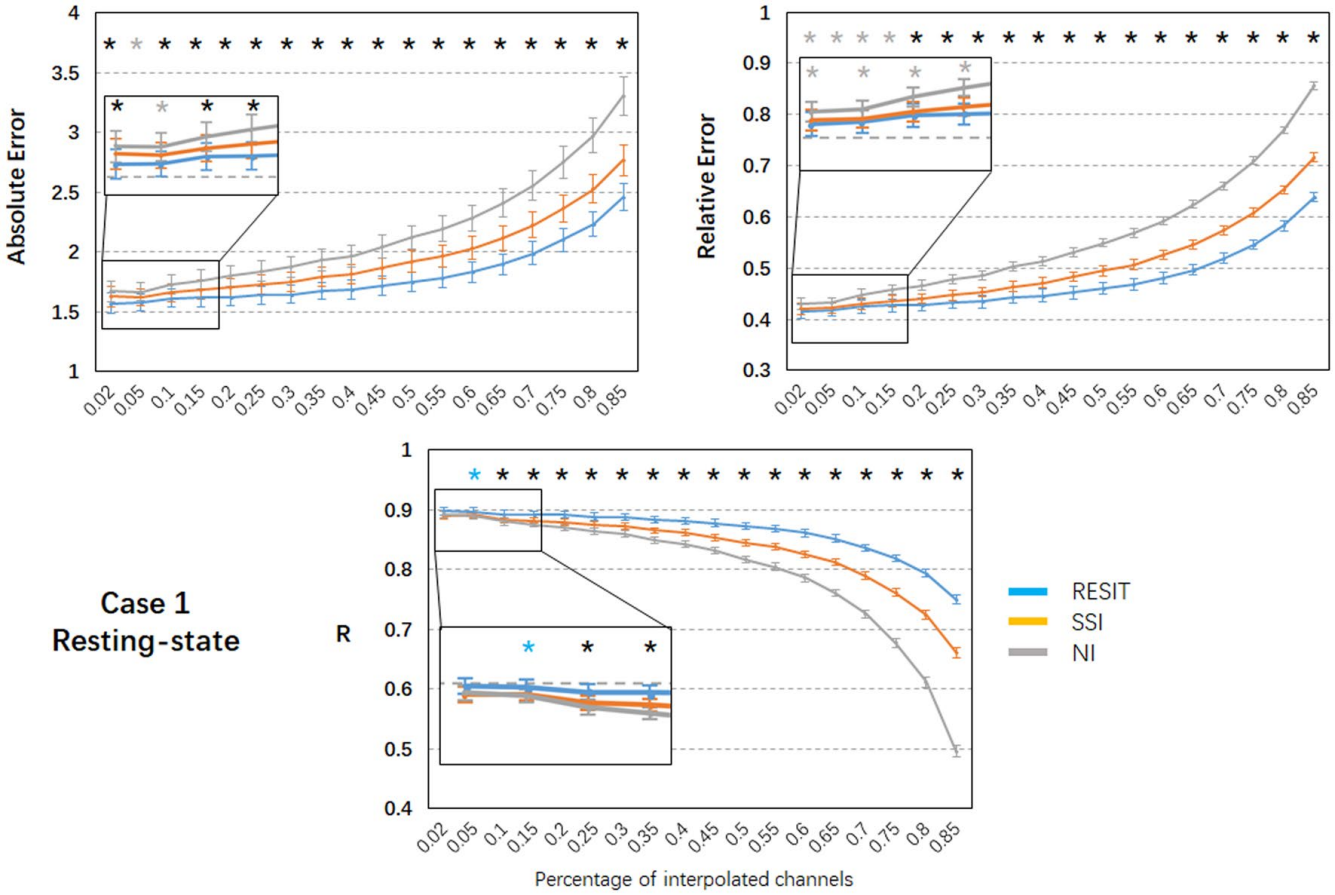
Performance of the RESIT, SSI and NI interpolation methods for resting-state EEG data in case 1. Mean values (absolute error, relative error and correlation) with standard error across subjects are shown. Black * indicates significant differences among the RESIT, SSI and NI methods; blue * indicates significant differences between the RESIT and SSI/NI methods; gray * indicates significant differences between the NI and RESIT/SSI methods; significance was set at *P* < 0.005; R: correlation (Color figure online)

**Fig. 4:**
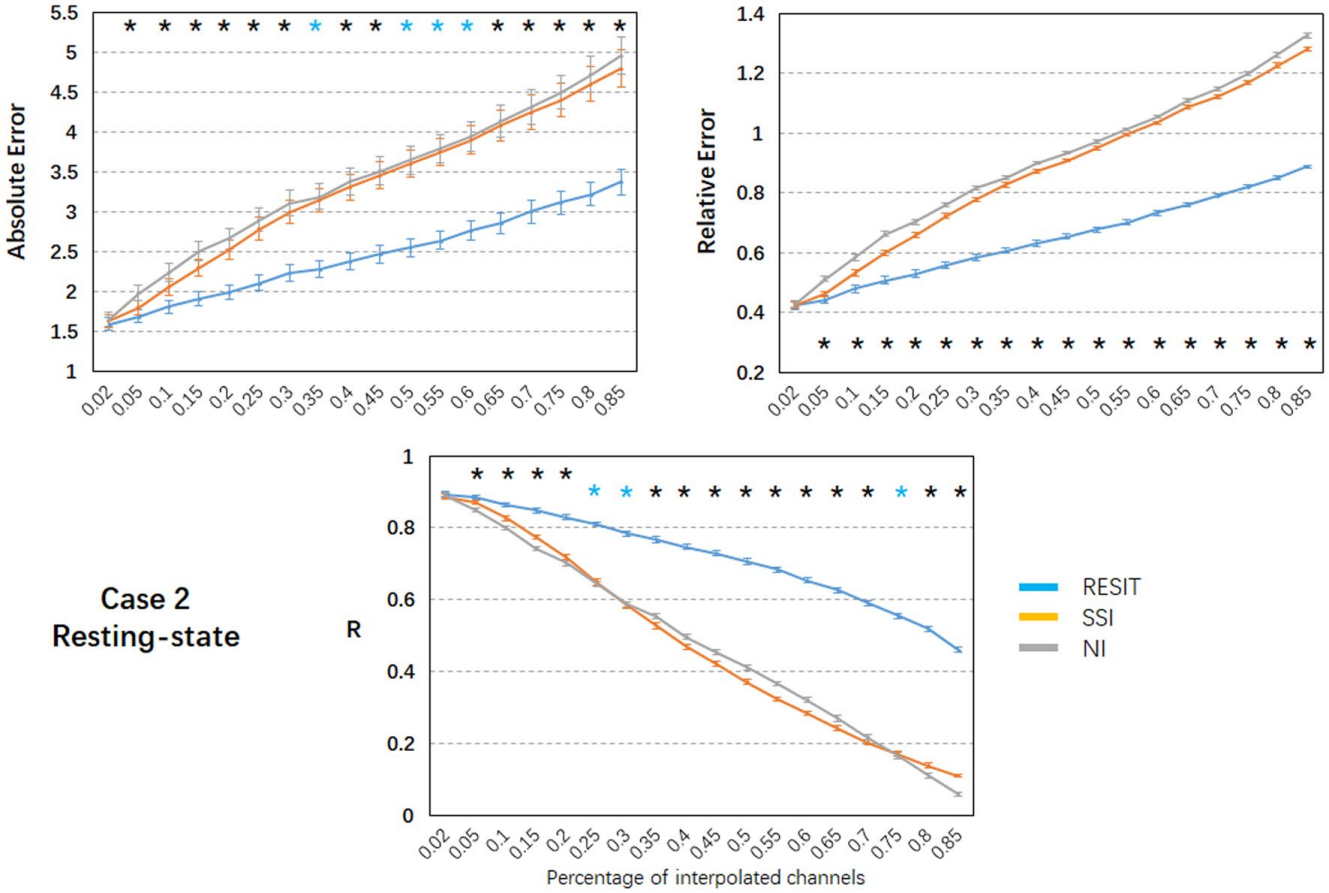
Performance of the RESIT, SSI and NI interpolation methods for resting-state EEG data in case 2. Mean values (absolute error, relative error and correlation) with standard error across subjects are shown. Black * indicates significant differences among the RESIT, SSI and NI methods; blue * indicates significant differences between the RESIT and SSI/NI methods; significance was set at *P* < 0.005, R: correlation (Color figure online)

Figure 2 depicts the signal waves and topographic maps of the resting-state EEG data for true signals and those interpolated from the RESIT, SSI and NI methods from a same example subject. A visual inspection of the results showed that, assuming 10% bad channels, all methods can reconstruct the bad channels for cases 1 and 2 to some degree. To quantify the performances of the interpolation methods, the absolute error, relative absolute error and correlation (R) were further calculated for different percentages of bad channels, and the mean results across subjects are shown in Figs. 3–4. In case 1 (Fig. 3), as the percentage of bad channels increased, the mean absolute error and relative absolute error of all methods generally increased, and the mean correlations of all methods decreased. Using one-way repeated ANOVA (*p* < 0.01) and post hoc paired t-test (*p* < 0.005), for the entire range of bad channel percentages (2% to 85%), almost all differences in the pairwise comparisons of the errors and R among these interpolation methods were significant. The RESIT introduced the smallest absolute errors, smallest relative errors and largest correlations, while SSI introduced intermediate errors and correlations, and NI had the largest errors and smallest correlations. Compared with SSI (Table S1), the RESIT resulted in an approximately 2.87% ~ 11.61% reduction in the absolute error, an approximately 0.78% ~ 10.95% reduction in the relative error, and an approximately 0.67% ~ 13.48% increase in the correlation. Compared with NI (Table S1), the RESIT resulted in an approximately 5.69% ~ 25.61% reduction in the absolute error, an approximately 3.24% ~ 25.49% reduction in the relative error, and an approximately 0.61% ~ 51.25% increase in the correlation.

In case 2 (Fig. 4), as the percentage of bad channels increased, the mean absolute error and relative absolute error of all methods linearly increased, and the mean correlations of all methods linearly decreased. Using one-way repeated ANOVA (*p* < 0.01) and post hoc paired t-test (*p* < 0.005), for the range of bad channel percentages (2% to 85%), almost all differences for the pairwise comparisons of the errors and R among these interpolation methods were significant. The RESIT introduced the smallest errors and largest correlation, while SSI and NI introduced similar errors and correlations to some degree. Compared with SSI (Table S2), the RESIT resulted in an approximately 2.39% ~ 29.98% reduction in the absolute error, an approximately 0.30% ~ 30.67% reduction in the relative error, and an approximately 0.73% ~ 320.74% increase in the correlation. Compared with NI (Table S2), the RESIT resulted in an approximately 3.36% ~ 31.99% reduction in the absolute error, an approximately 1.27% ~ 33.07% reduction in the relative error, and an approximately 0.36% ~ 691.02% increase in the correlation.

### P300 Data

**Fig. 5:**
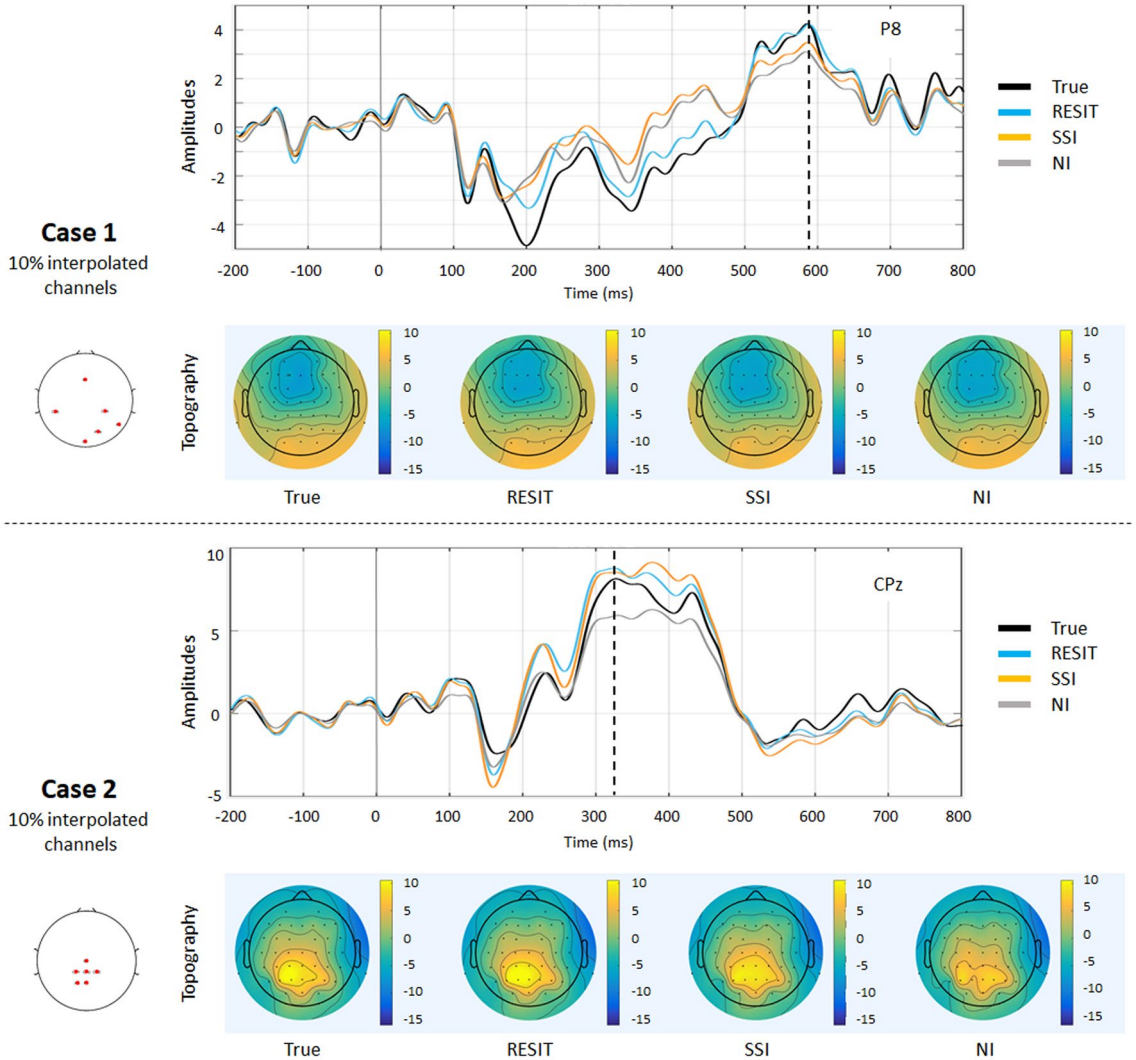
Results of the RESIT, SSI and NI interpolation methods for resting-state EEG data from one example subject. In case 1, a number of scattered bad channels (10%) were randomly assigned, and in case 2, a set of adjacent bad channels (10%) was randomly assigned

**Fig. 6:**
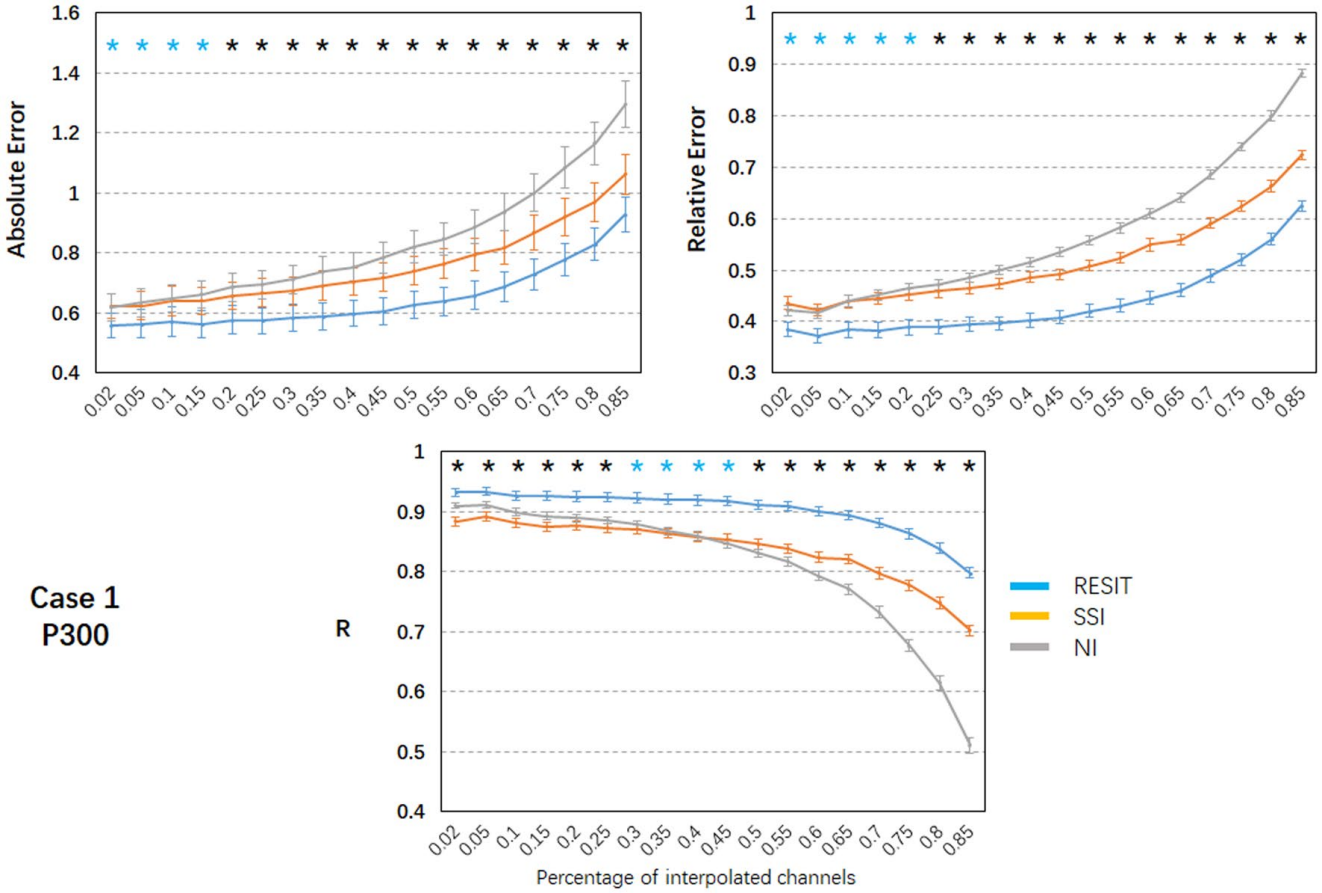
Performance of the RESIT, SSI and NI interpolation methods for the P300 EEG data in case 1. Mean values (absolute error, relative error and correlation) with standard error across subjects are shown. Black * indicates significant differences among the RESIT, SSI and NI methods; blue * indicates significant differences between the RESIT and SSI/NI methods; significance was set at *P* < 0.005; R: correlation (Color figure online)

Figure 5 depicts the ERP signal waves and topographic maps of the P300 data for the true signals and those interpolated with the RESIT, SSI and NI methods from a same example subject. Visual inspection of the results shows that for 10% bad channels, all methods can reconstruct the ERPs of the bad channels in cases 1 and 2 to some degree. In case 1 (Fig. 6), as the percentage of bad channels increased, the mean absolute error and relative absolute error of all methods generally increased, and the mean correlations of all methods decreased. Using one-way repeated ANOVA (*p* < 0.01) and post hoc paired t-test (*p* < 0.005), for the range of bad channel percentages from 2% to 85%, significant differences for the pairwise comparisons of the errors and R of the ERPs were found among these interpolation methods. The RESIT introduced the smallest absolute error, smallest relative error and largest correlations. SSI introduced intermediate absolute and relative errors for 25% ~ 85% bad channels and intermediate correlations for 50% ~ 85% bad channels. NI had the largest errors for 25% ~ 85% bad channels, intermediate correlations for 2% ~ 25% bad channels and the smallest correlations for 50% ~ 85% bad channels. Compared with SSI (Table S3), the RESIT resulted in an approximately 9.70% ~ 17.05% reduction in the absolute error, an approximately 11.60% ~ 18.82% reduction in the relative error, and an approximately 4.71% ~ 13.61% increase in the correlation. Compared with NI (Table S3), the RESIT resulted in an approximately 9.98% ~ 28.82% reduction in the absolute error, an approximately 8.68% ~ 30.01% reduction in the relative error, and an approximately 2.41% ~ 56.42% increase in the correlation.

**Fig. 7:**
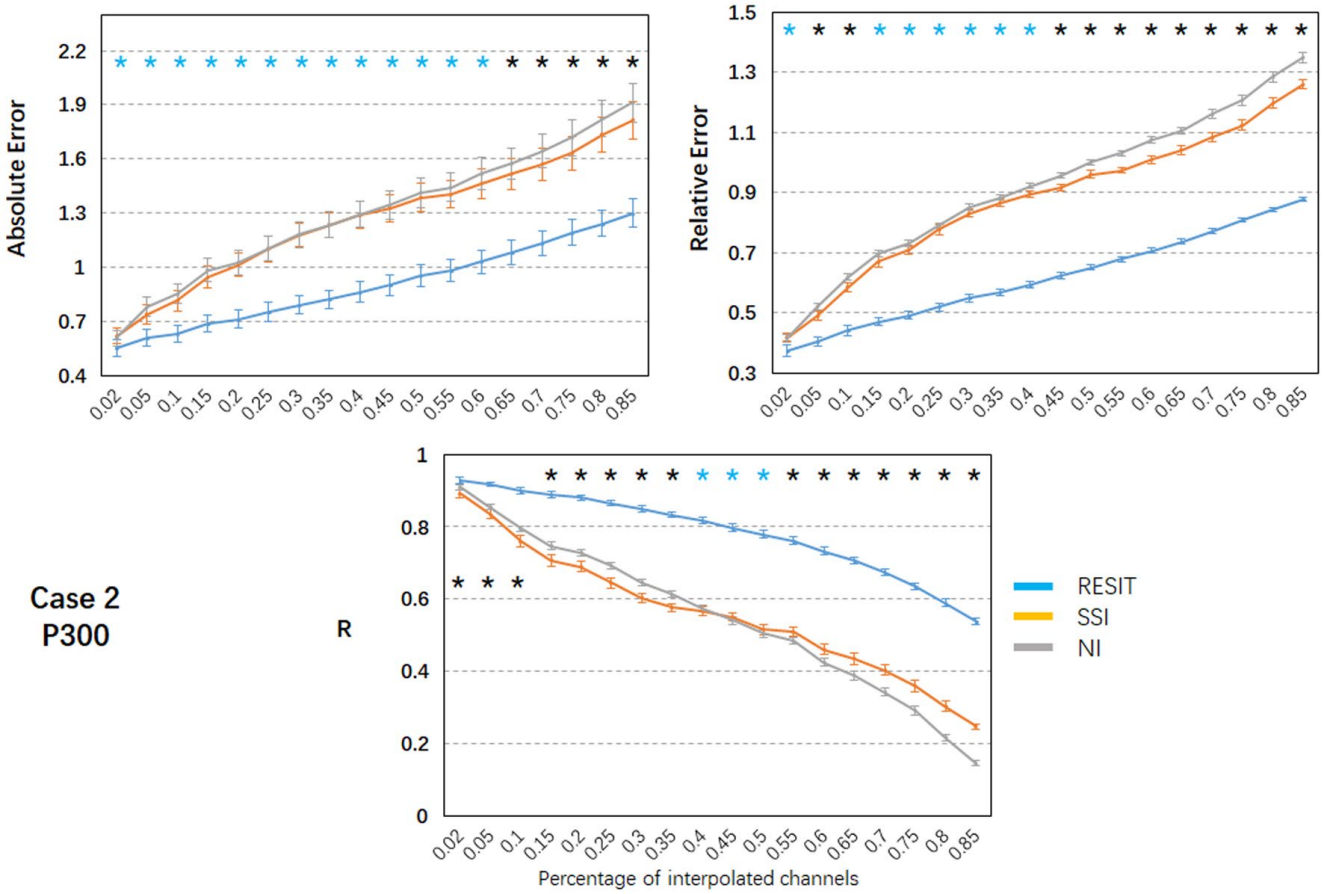
Performance of the RESIT, SSI and NI interpolation methods for the P300 EEG data in case 2. Mean values (absolute error, relative error and correlation) with standard error across subjects are shown. Black * indicates significant differences among the RESIT, SSI and NI methods; blue * indicates significant differences between the RESIT and SSI/NI methods; significance was set at *P* < 0.005; R: correlation (Color figure online)

In case 2 (Fig. 7), the mean absolute error and relative absolute error of all methods linearly increased as the percentage of bad channels increased, and the mean correlations of all methods linearly decreased with increases in the percentage of bad channels. Using one-way repeated ANOVA (*p* < 0.01) and post hoc paired t-test (*p* < 0.005), for the range of bad channel percentages from 2% to 85%, significant differences for the pairwise comparisons of the errors and R of the ERPs were found among these interpolation methods. The RESIT introduced the smallest absolute and relative errors and largest correlations. SSI introduced intermediate absolute errors for 65% ~ 85% bad channels, intermediate relative errors for 45% ~ 85% bad channels, and intermediate correlations for 55% ~ 85% bad channels. NI resulted in intermediate correlations for 2% ~ 35% bad channels. Compared with SSI (Table S4), the RESIT resulted an approximately 10.42% ~ 33.49% reduction in the absolute error, an approximately 10.74% ~ 34.39% reduction in the relative error, and an approximately 4.18% ~ 117.84% increase in the correlation. Compared with NI (Table S4), the RESIT resulted in an approximately 8.61% ~ 33.48% reduction in the absolute error, an approximately 10.17% ~ 35.70% reduction in the relative error, and an approximately 1.98% ~ 270.14% increase in the correlation.

## Discussion

In the present study, a new interpolation method, named RESIT, was proposed to reconstruct bad scalp EEG channels. Two cases involving bad channels were implemented to assess the performance of the RESIT as well as of common interpolation methods including NI and SSI.

### Methodological Considerations of the RESIT

To illustrate the performance of the RESIT, a resting-state EEG dataset was first used to obtain the results. In both case 1 with 10% scattered bad channels and case 2 with 10% adjacent bad channels, the RESIT performed well in reconstructing the scalp EEG signals (Fig. 2). Using a P300 dataset, the RESIT still performed well in reconstructing ERPs in both cases 1 and 2 (Fig. 5). It is not uncommon that there may be a number of bad channels during recordings for various reasons, such as power line interference (50/60 Hz), abnormal impedance, broken wire contacts, improperly placed or bridged channels, etc. (Hu and Zhang 2019). Therefore, it is necessary to reconstruct these bad channels when preprocessing EEG data. For traditional methods such as NI and SSI (Freeden 1984; Perrin et al. 1989), the transform matrices (Eqs. 1–2) that describe the relationship from the good channels to the interpolated channels are just mathematical operations that represent nonphysical principle-based hypotheses (NI: averaging; SSI: interpolation using spherical splines). According to Eqs. (7–8), the RESIT is based on the equivalent sources model (Geselowitz 1998; Pascualmarqui and Lehmann 1993), head model and electrode montage, and the transform matrix $L\tilde{L}^{ + }$ that describes the relationship from the good channels to the interpolated channels is physically based and reasonable. The estimated potentials $\hat{V}_{Inf}$ in Eq. (8), which contain all the good and interpolated channels, are technically converted to use a reference at infinity at the same time. That is, the RESIT has the ability to approximately convert an average or unipolar reference to an ideal zero reference. To some degree, the RESIT may address the fact that in the scalp EEG domain, there is no point on the body or head that could be used as an ideal reference with zero or constant potential (Luck 2014; Yao et al. 2019). It is worth noting that if there are no bad channels, $\hat{V}_{Inf}$ is absolutely equivalent to scalp EEG signals with an infinity reference realized by the REST (Dong et al. 2017; Yao 2001; Yao et al. 2005). If there are *k* bad channels, the realizations of RESIT and REST are different. However, considering the extrapolation itself in the realization, the current RESIT approach may be presented as a generalization of REST, conceptually. In both cases, as the percentage of bad channels increased, the absolute error and relative absolute error of the RESIT generally increased, and the correlations decreased, both for the resting-state EEG data (Figs. 3–4) and for the ERPs (Figs. 6–7). These results demonstrate that the performance of RESIT could benefit from a large number of good channels for both resting-state and P300 EEG datasets. Meanwhile, the performance of the RESIT could suffer when there is a large number of adjacent bad channels. Overall, the more good channels and the fewer adjacent bad channels there are, the more accurate the interpolation of EEG data for the bad channels will be using the RESIT.

### Comparison with NI and SSI

To further investigate the superiority of the RESIT, the indices of errors and correlations of NI and SSI were calculated and compared with those of the RESIT. Using one-way repeated ANOVA (*p* < 0.01) and post hoc paired t-test (*p* < 0.005), for the range of bad channel percentages (2% to 85%), there were significant differences in the indices among these interpolation methods (Figs. 3, 4, 6 and 7). For both the resting-state and P300 datasets, as a simple interpolation method, NI produced nearly all the largest errors and smallest correlation for both cases 1 and 2 (NI only slightly outperformed SSI only for some percentages of bad channels). NI produced satisfactory performance for a few scattered bad channels only, and as the percentage of bad channels increased, especially for the adjacent bad channels case (case 2), the method became more unsuitable. SSI, another common interpolation method in the EEG domain (Freeden 1984; Perrin et al. 1989), uses the spherical spline to interpolate bad channels based on the fact that the EEG electrode distribution of the cap system could be projected to a sphere. Overall, its performance was better than that of NI; however, it was worse than the RESIT. Because the nonphysical natures of SSI (Freeden 1984; Perrin et al. 1989) and NI require a sufficient number of valid adjacent channels, their potential to efficiently use the information from the good channels to reconstruct the EEG signals of the bad channels is limited, especially when there are many adjacent bad channels. To some degree, the RESIT could tolerate increases in the number of scattered and adjacent bad channels due to its physical equivalent source estimating and forwarding ability (Yao 2000, 2001). Furthermore, in case 2, the correlations between the RESIT-interpolated and true signals were higher than those of NI and SSI (except for approximately 30%-40% adjacent bad channels, for which the correlations were approximately 0.8). This implies that for a number of adjacent bad channels, compared with those of traditional interpolation methods NI and SSI, the EEG signals interpolated by the RESIT may be more suitable for constructing EEG networks for assessing functional connectivity of the brain via signal synchronization (Jalili et al. 2014; Li et al. 2019, 2015; Xu et al. 2014).

### Benefits and perspectives on the RESIT

Current interpolation methods always directly utilize interpolation theory to reconstruct the scalp EEG signals of bad channels, ignoring the neurophysiological basis of the generation of EEG signals. Based on physical principle-based hypotheses, the RESIT can quickly and effectively reconstruct the signals of bad channels accompanied by rereferencing to an ideal infinity reference. Therefore, the RESIT may have better performance than current methods, such as NI and SSI. As a novel interpolation method, the RESIT has been integrated in the EEG preprocessing pipeline on the WeBrain cloud platform (https://webrain.uestc.edu.cn/), and it is further expected to be integrated into common EEG tools such as EEGLAB (Delorme and Makeig 2004) and FieldTrip (Oostenveld et al. 2011), as well as other EEG preprocessing pipelines and tools for simultaneous EEG-fMRI multimodal fusion (Dong et al. 2014, 2018) and large-scale EEG preprocessing. In addition, because no interpolation method provides completely unique data for the missing channels, the interpolated channels using the RESIT are not independent, which may reduce the spatial resolution of the EEG when there are too many bad channels.

## Conclusions

In conclusion, the novelty of this work is the interpolation process termed the RESIT, in which we utilize the classical equivalent source technique and forward approach to model the generation of scalp EEG signals with an ideal infinity reference, which was demonstrated with resting-state and event-related EEG datasets. The proposed RESIT method performs better (lower errors and higher correlations) than traditional methods such as NI and SSI and has the potential for applications in separate and simultaneous EEG preprocessing, which would benefit further EEG analysis, including ERP analysis, EEG network analysis, and strict group-level statistics.

## Data and Code Availability

The datasets and codes used in this study are available on reasonable request to the corresponding author.

## Supplementary Information

Below is the link to the electronic supplementary material.

Supplementary file1 (DOCX 19 kb)
