# Supplementary material for: Reference Electrode Standardization Interpolation Technique (RESIT): A Novel Interpolation Method for Scalp EEG
Source: Brain Topogr. 2021 May 5;34(4):403–14. doi: 10.1007/s10548-021-00844-2 (PMC8195908; doi:10.1007/s10548-021-00844-2)
Supplement: Supplementary file 1 — Supplementary file1 (DOCX 19 kb) [file 10548_2021_844_MOESM1_ESM.docx]

Supplementary Materials

Table S1: The performance of RESIT, compared with SSI and NI methods for resting-state data in case 1. -: reduction; +: increase.

| **Resting-state**  **Case 1** | RESIT vs SSI | | | RESIT vs NI | | |
| --- | --- | --- | --- | --- | --- | --- |
| Percentage of interpolated channels | Absolute Error (%) | Relative Absolute Error(%) | R(%) | Absolute Error(%) | Relative Absolute Error(%) | R(%) |
| 2% | -3.54 | -1.16 | +0.75 | -5.88 | -3.36 | +0.61 |
| 5% | -2.90 | -0.78 | +0.67 | -5.69 | -3.24 | +0.82 |
| 10% | -2.87 | -1.02 | +0.98 | -6.45 | -4.80 | +1.41 |
| 15% | -3.98 | -1.93 | +1.18 | -8.34 | -6.64 | +1.94 |
| 20% | -4.73 | -2.71 | +1.47 | -9.79 | -8.17 | +2.48 |
| 25% | -5.07 | -3.05 | +1.57 | -10.69 | -9.10 | +2.81 |
| 30% | -6.02 | -4.09 | +1.83 | -12.08 | -10.63 | +3.34 |
| 35% | -6.59 | -4.56 | +2.11 | -13.36 | -11.85 | +4.06 |
| 40% | -7.09 | -5.28 | +2.40 | -14.35 | -13.09 | +4.70 |
| 45% | -8.17 | -6.49 | +2.89 | -16.02 | -14.77 | +5.65 |
| 50% | -8.83 | -7.04 | +3.29 | -17.44 | -16.23 | +6.90 |
| 55% | -9.09 | -7.42 | +3.56 | -18.51 | -17.49 | +7.95 |
| 60% | -9.97 | -8.55 | +4.25 | -19.81 | -18.85 | +9.52 |
| 65% | -10.40 | -9.04 | +4.93 | -21.26 | -20.47 | +12.03 |
| 70% | -10.61 | -9.55 | +5.99 | -22.11 | -21.50 | +15.27 |
| 75% | -11.19 | -10.29 | +7.52 | -23.70 | -23.13 | +20.91 |
| 80% | -11.61 | -10.81 | +9.65 | -24.85 | -24.31 | +29.76 |
| 85% | -11.13 | -10.95 | +13.48 | -25.61 | -25.49 | +51.25 |

Table S2: The performance of RESIT, compared with SSI and NI methods for resting-state data in case 2. -: reduction; +: increase.

| **Resting-state**  **Case 2** | RESIT vs SSI | | | RESIT vs NI | | |
| --- | --- | --- | --- | --- | --- | --- |
| Percentage of interpolated channels | Absolute Error (%) | Relative Absolute Error(%) | R(%) | Absolute Error(%) | Relative Absolute Error(%) | R(%) |
| 2% | -2.39 | -0.30 | +0.73 | -3.36 | -1.27 | +0.36 |
| 5% | -6.28 | -3.85 | +1.52 | -14.62 | -13.30 | +4.09 |
| 10% | -12.04 | -9.68 | +4.55 | -19.09 | -17.94 | +8.03 |
| 15% | -17.09 | -15.44 | +9.51 | -23.78 | -23.36 | +14.27 |
| 20% | -20.98 | -19.48 | +15.50 | -25.01 | -24.62 | +18.28 |
| 25% | -24.29 | -22.95 | +24.65 | -27.14 | -26.86 | +25.65 |
| 30% | -25.60 | -24.67 | +34.02 | -28.34 | -28.27 | +33.66 |
| 35% | -27.42 | -26.58 | +45.54 | -28.42 | -28.58 | +38.64 |
| 40% | -28.38 | -27.68 | +59.34 | -29.78 | -29.93 | +50.49 |
| 45% | -28.51 | -28.15 | +73.53 | -29.68 | -30.11 | +60.88 |
| 50% | -29.03 | -28.75 | +91.21 | -29.98 | -30.37 | +71.57 |
| 55% | -29.54 | -29.49 | +112.02 | -30.29 | -30.71 | +86.84 |
| 60% | -29.15 | -29.20 | +130.99 | -29.92 | -30.45 | +104.16 |
| 65% | -29.98 | -29.96 | +159.70 | -30.90 | -31.31 | +132.91 |
| 70% | -29.34 | -29.50 | +192.12 | -30.39 | -30.95 | +173.85 |
| 75% | -29.20 | -29.75 | +226.59 | -30.68 | -31.48 | +236.20 |
| 80% | -29.97 | -30.67 | +275.79 | -31.74 | -32.63 | +368.12 |
| 85% | -29.64 | -30.65 | +320.74 | -31.99 | -33.07 | +691.02 |

Table S3: The performance of RESIT, compared with SSI and NI methods for P300 data in case 1. -: reduction; +: increase.

| **P300**  **Case 1** | RESIT vs SSI | | | RESIT vs NI | | |
| --- | --- | --- | --- | --- | --- | --- |
| Percentage of interpolated channels | Absolute Error (%) | Relative Absolute Error(%) | R(%) | Absolute Error(%) | Relative Absolute Error(%) | R(%) |
| 2% | -10.47 | -11.60 | +5.43 | -9.98 | -8.68 | +2.41 |
| 5% | -9.70 | -11.99 | +4.71 | -11.18 | -10.74 | +2.48 |
| 10% | -10.76 | -12.61 | +5.13 | -11.78 | -12.87 | +3.06 |
| 15% | -12.08 | -14.21 | +5.83 | -14.84 | -15.38 | +3.74 |
| 20% | -12.15 | -14.20 | +5.47 | -15.84 | -16.28 | +4.00 |
| 25% | -13.79 | -15.13 | +6.01 | -17.25 | -17.48 | +4.49 |
| 30% | -13.13 | -15.19 | +5.92 | -17.94 | -18.81 | +5.06 |
| 35% | -14.93 | -16.34 | +6.53 | -20.28 | -20.82 | +6.15 |
| 40% | -15.05 | -17.19 | +7.16 | -20.53 | -21.87 | +6.92 |
| 45% | -15.46 | -17.02 | +7.41 | -22.69 | -23.67 | +8.32 |
| 50% | -15.35 | -17.21 | +7.77 | -23.54 | -24.58 | +9.74 |
| 55% | -16.53 | -17.81 | +8.37 | -24.63 | -26.00 | +11.28 |
| 60% | -17.05 | -18.82 | +9.42 | -25.66 | -27.05 | +13.61 |
| 65% | -16.21 | -17.53 | +9.03 | -26.76 | -28.08 | +16.02 |
| 70% | -16.17 | -17.32 | +10.46 | -27.13 | -28.75 | +20.31 |
| 75% | -15.45 | -16.67 | +11.11 | -28.30 | -29.68 | +27.60 |
| 80% | -14.50 | -15.67 | +12.18 | -28.82 | -30.01 | +36.50 |
| 85% | -12.67 | -13.69 | +13.61 | -28.39 | -29.25 | +56.42 |

Table S4: The performance of RESIT, compared with SSI and NI methods for P300 data in case 2. -: reduction; +: increase.

| **P300**  **Case 2** | RESIT vs SSI | | | RESIT vs NI | | |
| --- | --- | --- | --- | --- | --- | --- |
| Percentage of interpolated channels | Absolute Error (%) | Relative Absolute Error(%) | R(%) | Absolute Error(%) | Relative Absolute Error(%) | R(%) |
| 2% | -10.42 | -10.74 | +4.18 | -8.61 | -10.17 | +1.98 |
| 5% | -17.70 | -17.31 | +9.91 | -22.01 | -22.30 | +7.44 |
| 10% | -22.71 | -24.72 | +18.31 | -26.22 | -28.72 | +13.12 |
| 15% | -27.44 | -29.91 | +25.78 | -30.21 | -32.55 | +18.99 |
| 20% | -29.89 | -30.70 | +27.82 | -30.75 | -32.73 | +20.80 |
| 25% | -31.54 | -33.19 | +34.42 | -31.62 | -34.36 | +24.98 |
| 30% | -32.83 | -34.06 | +40.59 | -32.98 | -35.56 | +31.56 |
| 35% | -33.49 | -34.39 | +44.77 | -33.48 | -35.70 | +36.15 |
| 40% | -33.04 | -33.47 | +44.61 | -33.27 | -35.38 | +42.62 |
| 45% | -32.15 | -31.94 | +44.92 | -33.02 | -34.87 | +47.70 |
| 50% | -31.43 | -32.18 | +50.98 | -32.76 | -34.86 | +54.12 |
| 55% | -29.84 | -30.27 | +50.09 | -31.87 | -34.14 | +57.71 |
| 60% | -29.79 | -30.02 | +58.94 | -32.32 | -34.28 | +72.41 |
| 65% | -28.62 | -29.17 | +62.68 | -31.18 | -33.30 | +82.15 |
| 70% | -28.08 | -28.71 | +67.49 | -31.26 | -33.51 | +97.49 |
| 75% | -26.89 | -27.99 | +76.03 | -30.54 | -33.04 | +117.80 |
| 80% | -28.38 | -29.54 | +94.76 | -31.89 | -34.31 | +173.21 |
| 85% | -28.34 | -30.26 | +117.84 | -31.97 | -34.86 | +270.14 |
